# Facilitation of information processing in the primary somatosensory area in the ball rotation task

**DOI:** 10.1038/s41598-017-15775-x

**Published:** 2017-11-14

**Authors:** Toshiaki Wasaka, Tetsuo Kida, Ryusuke Kakigi

**Affiliations:** 1 Department of Engineering, Nagoya Institute of Technology, Nagoya, Japan; 2 Department of Integrative Physiology, National Institute for Physiological Sciences, Okazaki, Japan

## Abstract

Somatosensory input to the brain is known to be modulated during voluntary movement. It has been demonstrated that the response in the primary somatosensory cortex (SI) is generally gated during simple movement of the corresponding body part. This study investigated sensorimotor integration in the SI during manual movement using a motor task combining movement complexity and object manipulation. While the amplitude of M20 and M30 generated in the SI showed a significant reduction during manual movement, the subsequent component (M38) was significantly higher in the motor task than in the stationary condition. Especially, that in the ball rotation task showed a significant enhancement compared with those in the ball grasping and stone and paper tasks. Although sensorimotor integration in the SI generally has an inhibitory effect on information processing, here we found facilitation. Since the ball rotation task seems to be increasing the demand for somatosensory information to control the complex movements and operate two balls in the palm, it may have resulted in an enhancement of M38 generated in the SI.

## Introduction

Voluntary movement activates proprioceptive and cutaneous receptors, and somatosensory information from moving body parts provide the status of movement in a moment. Preceding and during movement, the motor and higher-order brain systems regulate sensory inflow during its course from the peripheral to the somatosensory areas. Using electroencephalography and magnetoencephalography, we can investigate information processing in somatosensory areas. The amplitude of short-latency somatosensory evoked potentials (SEPs) and somatosensory evoked magnetic fields (SEFs) components generated in the primary somatosensory cortex (SI) is diminished when a stimulus is delivered to the peripheral nerve innervating the contracting muscle. This phenomenon is called ‘gating’. It is considered that the functional role of this phenomenon is to regulate the inflow of somatosensory information so as not to induce an unintended reflex by somatosensory feedback.

The amplitude of SEPs/SEFs during voluntary movement is generally depressed compared with those in a stationary condition, and the gain reduction of SEPs/SEFs was dependent on the parameters of muscle contraction such as the level of contractile force^1–4^ or contraction velocity^5–7^. However, to investigate sensorimotor integration in somatosensory areas, previous studies mainly used a simple movement such as hand gripping^8^, finger extension/flexion of the upper limb^9–11^, or plantar flexion/dorsiflexion of the lower limb^11,12^. Previous studies showed that the pattern of cortical activation in sensorimotor areas is different between simple and complex finger movements in normal^13^ and dystonia patients^14,15^. In addition, movement-induced modulation of SEPs in upper limb dystonia showed abnormal sensorimotor integration in somatosensory areas^16,17^. Therefore, activation in the somatosensory area during complex movement needs to be further investigating for greater understanding of the neural mechanisms of sensorimotor integration.

Manual movement in daily life requires coordinated or independent finger movement. This ability of fingers allows us to operate objects using fingers dexterously. The purpose of this study was to identify whether movement-induced gain modulation in somatosensory areas differed in dexterous finger movements. The present study used four active motor tasks combining movement complexity of fingers and manipulation of objects. The motor tasks were the rotation of two balls in the right palm (the BR task), ball grasping with the right hand (the BG task), opening and closing the right hand continuously (the SP task), and movement of the right fingers as if subjects were performing the BR task in the absence of the two balls (the AR task).

We previously reported that the modulation of SEPs/SEFs components during movement execution was not a simple inhibition, but information processing in the SI varied depending on the context of muscle contraction^8^. The early SEP components were significantly elevated when subjects were required to track the continuous passive movement of one foot actively with the opposite foot, compared to those recorded during passive ankle plantarflexion and dorsiflexion^18^. Since our fingers work as sensory and motor organs, we hypothesized that sensory demands for somatosensory information to execute motor tasks modulates information processing in the SI.

## Results

**Figure 1:**
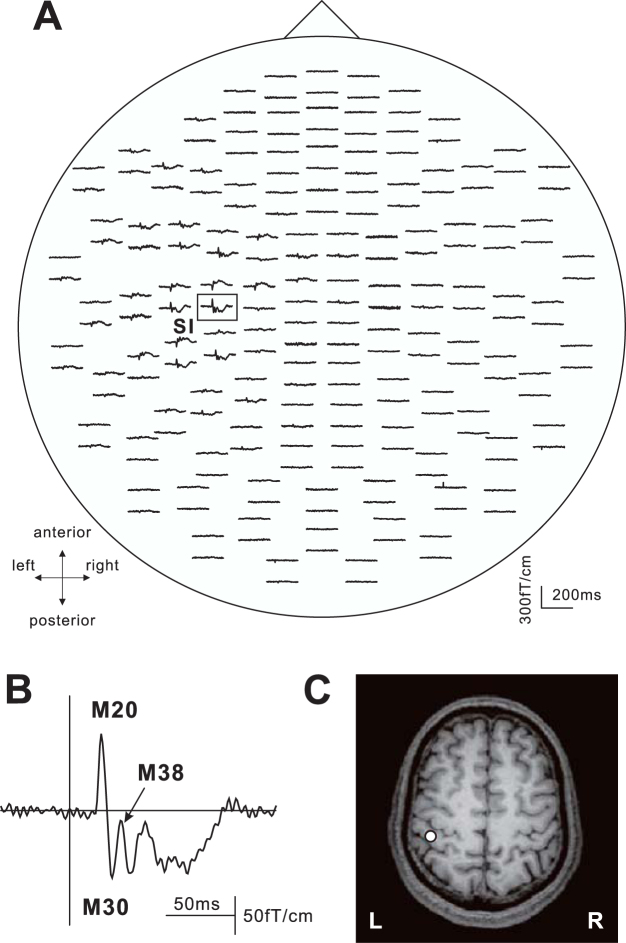
Somatosensory evoked magnetic fields following stimulation of the right median nerve in a subject. (**A**) The 204-channnel SEF waveforms in a stationary condition viewed from the top of the head. Clear deflections were obtained in the central region contralateral to the side of simulation. (**B**) An enlarged waveform obtained from one gradiometer channel in the SI (framed channel in A). A vertical line indicates the onset of electrical stimulation. (**C**) The location of an equivalent current dipole (ECD) in M20 superimposed on the 2D image. The ECD was located in the posterior bank of the central sulcus in the hemisphere contralateral to the simulated side.

**Figure 2:**
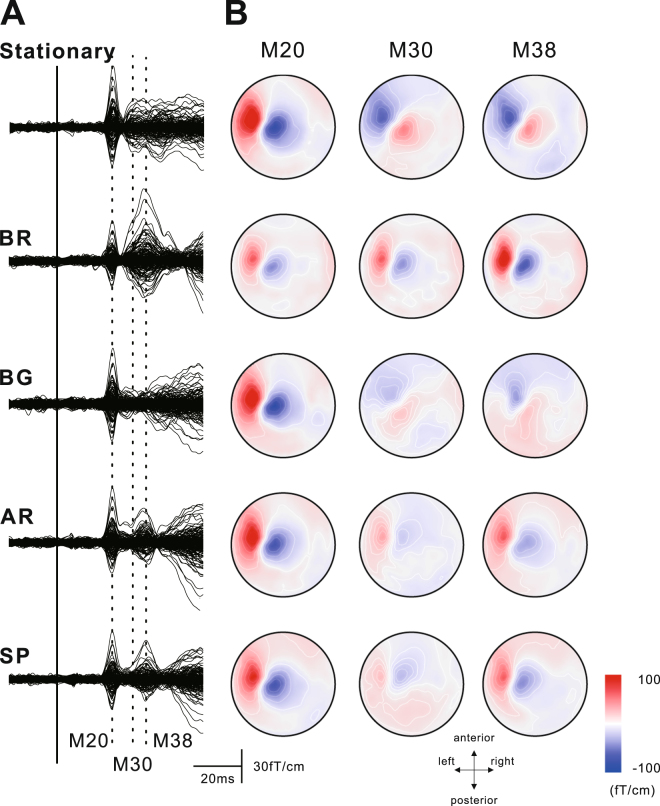
Grand averaged waveforms of the superimposed SEFs (10 subjects) in stationary and four motor conditions (**A**) and topographical maps of M20, M30, and M38 components (**B**). The artifact following electrical stimulation was eliminated in off-line analysis. The motor tasks were the ball rotation task (BR), the ball grasping task (BG), the air rotation task (AR), and the stone and paper task (SP). In all conditions, the first deflection peaking around 20 ms (M20) following stimulation was observed. While the subsequent component (M30) was clearly identified under the stationary condition, those in the other conditions were decreased. In contrast, especially in the BR task, the component peaking around (M38) increased. Whereas the topographical map of the M20 component was the same in all conditions, that of M38 showed pattern reversal between the stationary condition and BR tasks.

**Figure 3:**
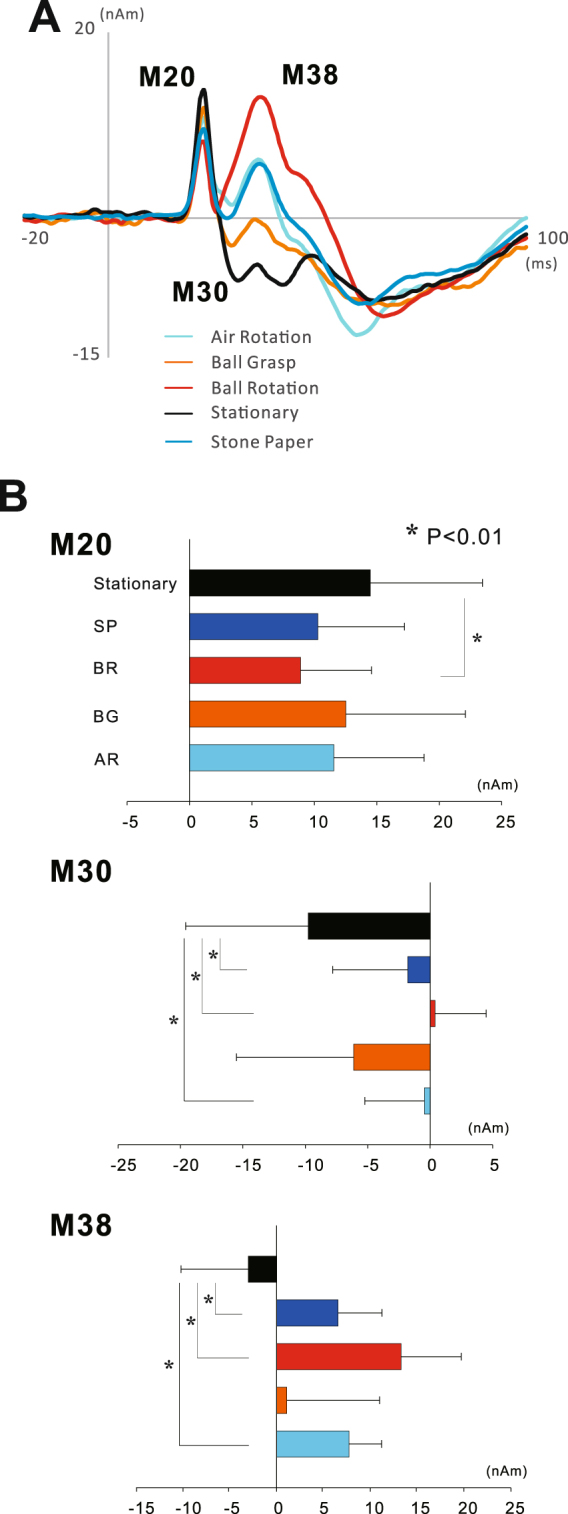
(**A**) The temporal change of the grand averaged ECD waveforms of the SI (10 subjects) in the stationary and four motor tasks. (**A**) Whereas the peak amplitude of M30 in four motor tasks decreases with motor tasks compared to the stationary condition, that of M38 enhanced during motor execution. (**B**) Mean amplitude of the ECD components of the SI. Vertical lines indicate standard deviations. Statistical significance compared with the stationary condition and each component showed that the peak amplitude of the ECD moment for M20 in the BR task was significantly smaller than that in the stationary condition. While, those for M30 in the BR, SP, and AR tasks were significantly smaller than the stationary condition, those for M38 showed a significant enhancement in the same tasks.

All subjects performed the four kinds of movement successfully and clear SEFs were recorded for all of them. Figure 1 shows the SEF waveforms following stimulation of the right median nerve in one subject. Clear components were obtained from the gradiometers of the central region in the hemisphere contralateral to the stimulated side at a latency less than 40 ms. The first deflection peaking at around 20 ms following stimulation (M20) and subsequent deflection peaking at around 30 ms (M30) were identified in the central region contralateral to the stimulated side. In addition, small peaks, about 40 ms (M38), appeared. The ECD for the first response was located in the posterior bank of the central sulcus corresponding to the SI. The superimposed SEF waveforms and topography of each component are shown in Fig. 2. The waveforms in the BR task appeared as a large deflection around 38 ms. A clear dipolar field pattern was observed in three components. However, whereas the topographical patterns in five experimental conditions were the same in M20, those of M30 and M38 showed a different pattern. In the stationary and BR tasks the pattern was reversed. Figure 3 shows the grand-averaged ECD waveforms of the SI in the stationary and four motor tasks and mean amplitude of ECD components.

First, to investigate the modulation of each component in four motor tasks compared to the stationary condition, we used a one-way repeated measures ANOVA. The results showed that a significant main effect was found for the peak ECD moment for M20 (F_(4,36)_ = 4.412, P < 0.05, ε = 0.468), M30 (F_(4,36)_ = 11.035, P < 0.01, ε = 0.347), and M38 (F_(4,36)_ = 19.728, P < 0.01, ε = 0.499). Post-hoc tests showed that the ECD moment for M20 was significantly smaller in the BR task than the stationary condition (P < 0.01). That for M30 was significantly smaller in the BR (P < 0.01), AR (P < 0.01) and SP tasks (P < 0.01) than in the stationary task. In contrast, that for M38 was significantly higher in the BR (P < 0.01), AR (P < 0.01) and SP tasks (P < 0.01) than in the stationary condition (Fig. 3).

Furthermore, to investigate which factors of finger movement influence sensorimotor integration in the SI, we used a two-way measures ANOVA with Movement Complexity and Object Operation as factors. A significant main effect was found for the ECD moment of M30 in Movement Complexity (F _(1,9)_ = 6.701, P < 0.05) and Object Operation (F _(1,9)_ = 8.671, P < 0.05), and that of M38 in Movement Complexity (F _(1,9)_ = 15.832, P < 0.01). In addition, a significant interaction was found between Movement Complexity and Object Operation for ECD moments of the M20 (F _(1,9)_ = 7.900, P < 0.05) and M38 (F _(1,9)_ = 24.299, P < 0.01). Post-hoc tests showed that the amplitude of M20 was significantly higher in the AR than in the BR task (P < 0.05), whereas there was no significant difference between the SP and BG tasks. Furthermore, that of M38 was significantly higher in the BR than in the AR task (P < 0.01), whereas it was significantly smaller in the SP than the BG task (P < 0.05).

## Discussion

This study showed different modulation of SEF components in the SI in relation to the types of finger movement. A reduction in the neuronal responses evoked by somatosensory stimulation during active movement has been reported at several levels in the central nervous system in animals, including the cuneate nucleus in cats^19^ and the spinal cord^20^ and SI in monkeys^21–23^ and human^9,24–26^. This phenomenon was observed as an attenuation of the short-latency SEPs/SEFs components and is called ‘gating’. Since our sensory systems are constantly bombarded by numerous sensory stimuli, the central nervous system must extract the few stimuli important for the control of movement. One can therefore recognize that an attenuation of SI activation is involved in filtering information. However, our results showed that the M38 component was significantly enhanced during the BR task, while other components, M20 and M30, showed significant reductions. The enhancement of M38 was somewhat unexpected, because modulation in the SI during movement is generally found to be inhibition. This result showed that sensorimotor integration in the SI is not a simple inhibitory effect.

### Attenuation of M20 and M30 during voluntary movement

Sensory input is known to strongly modulate motor output. Unregulated somatosensory inputs generated by movement might disturb motor execution by generating improper reafferent signals. Therefore, it is supposed that the central nervous system has a neural mechanism to inhibit incoming somatosensory information. For afferent information from the upper limb during movement, it is not clear if modulation occurs at a very early stage of information processing at the first cortical receptive site in area 3b and area 1. The change in the amplitude of M20, the earliest cortical response in the SI, with voluntary movement has been especially controversial. Some studies reported that the M20 did not change^10,27,28^, while others reported an attenuation in the amplitude^11,29^. In this study, a significant reduction in amplitude was found only in the BR task compared to the stationary condition, other motor tasks showed no changes in amplitude. In contrast, attenuation of the amplitude of M30 by voluntary movement has consistently been reported^10,11,27–29^. Our results showed that M30 was more sensitive to the effect of sensorimotor integration than M20. In general, the primary component (M20) is considered to be generated in Brodmann’s area 3b of the SI^30–33^. However, the generator for M30 remains unknown. Kawamura *et al*.^34^ suggested that area 4 of the primary motor cortex (MI) is involved in generating M30. Furthermore, Wikström *et al*.^35^, who investigated the modulation of SEF components caused by the effect of ISI, proposed that the mechanism responsible for P35m (corresponding to our M30) was inhibitory postsynaptic potentials in the deeper layers in area 3b. Lin *et al*.^36^ reported that M20 and M35 (corresponding to M30 in our study) showed distinctive neurophysiological behaviors in response to varying stimulus intensity levels. Although we could not show the temporal sequences of neural generators in the SI, the different modulation of M20 and M30 during voluntary movement may be caused by a difference in neural sources.

### Enhancement of M38 in the BR task

It is generally agreed that the modulation in short-latency SEPs/SEFs during voluntary movement is an inhibitory effect on the amplitude, but the result of our experiment showed that the BR task induced a significant enhancement of M38. The modulation of sensory processing in the somatosensory system could occur in two possible ways; one is interaction between the given sensory afferents for SEPs/SEFs and the afferent signals from the muscles, joints, and skin induced by the movement (centripetal gating), and the other is interaction between the given sensory signals and the efferent signals induced by the motor command from the motor-related areas (centrifugal gating)^37^. This study used four motor tasks combining the manipulation of objects and complex finger movement, and somatosensory feedback caused by finger movement occurred in all tasks. Furthermore, since cutaneous information generated by holding two balls also exists in the BR and BG tasks, the centripetal gating effect is acting on the SEF components in all tasks. Modulation of SEPs/SEFs caused by the centripetal gating mechanism using continuous tactile interference showed a reduction in amplitude^38^. However, the amplitude of M38 in the BR task showed a significant enhancement, not a reduction in other components in short-latency components, M20 and M30, reported in the previous studies. Therefore, there should be other neural mechanisms to cause this phenomenon.

One possibility for increasing M38 is the attentional effect. Typically, the attentional effect on SEP/SEF components is an enhancement, not a reduction. There are several reports showing the attentional effect on the short- and middle-latency SEP components that are generated in the SI^39–42^. However, subjects were instructed to concentrate on the finger movement and not to pay attention to the electrical stimulation during movement tasks. In addition, it is unlikely that attention to electrical stimulation affected the M38 only in the BR task. However, it is not possible to completely exclude the effect of attention on enhancement of M38 because attentional effects caused by the bimodal interaction of visual and somatosensory information modulate the SEP components. Similar P50 enhancement that was maximal with sensorimotor integration of task-relevant visual and tactile inputs has previously been reported^43^. Although it is difficult to compare the results of M38 following an electrical stimulation and the P50 component elicited by vibrotactile stimulation, there may be a similar neural mechanism that integrates visual and somatosensory information to control movement-enhanced activation of the SI. Another possibility is that the different sensory demands of motor tasks modulate somatosensory information processing by a centrifugal mechanism. The demand for somatosensory information needed to control sensory-guided behavior and exploration can enhance the amplitude of early SEP components^44,45^. The kinesthetic requirements to control movement execution modulated the amplitude of the SEP component by a centrifugal gating mechanism^18^. During rotation of two balls in the palm, since the subject is likely to concentrate on the moving fingers more than in the other task to conduct the dexterous motor task, the enhancement in the SEF component might have resulted from the fact that more somatosensory information from moving fingers must have been provided during this task.

We examined which factors of finger movement are acting on a facilitation of M38 using a two-way measures ANOVA, and the results showed a significant interaction between the movement complexity and object manipulation. This means that complex finger movement manipulating objects influenced the phenomenon in the BR task. The two balls were placed on the right palm and the subjects had to manipulate the objects with their right fingers. In this procedure, cutaneous and proprioceptive afferents as well as motor efferent signals were involved. Carefully controlled precision movement required sensory feedback and a specifically screened and selected afferent input may be necessary rather than just suppressing information at an early stage of information processing in the SI, because there is evidence for studies that at the cortical level also facilitation of certain somatosensory processing in the SI can occur during exploration of objects using fingers^44,46^.

The question to be solved is which kind of somatosensory input from the peripheral nerve is promoted in information processing in the SI during the BR task. For SEFs recording, we stimulated a median nerve that innervates the thumb, index, and middle fingers. Since the median nerve conveys mixed information from cutaneous and proprioceptive receptors, we could not confirm which somatosensory information induced the enhancement of M38. Confais *et al*.^47^ indicated that a facilitation of proprioceptive information processing in the SI occurs during voluntary movement, whereas cutaneous information processing resulted in an inhibition.

A previous study using dipole modeling on magnetic fields following electrical stimulation of the dorsum of the hand revealed a temporal relation of activities among multiple central cortical areas, Brodmann’s areas 3b, 4, 1, and the posterior parietal cortex around 20 to 40 ms^33^. In this study, although we tried to estimate the neural generator of M38 using multi-dipole modeling, we could not get a reliable model of temporal activation in multiple areas. Although the neural mechanism and origin of M38 were not clearly elucidated, we assumed that the M38 has a different component from M20 and M30 because of the different modulation in amplitudes.

In summary, we revealed that information processing in the SI during voluntary movement was not a simple inhibition of the incoming somatosensory feedback. Depending on the context of the motor task, it may occur as a facilitation, such as the observed enhancement of the SEFs component. Further studies will be needed to clarify the functional role of sensorimotor integration in somatosensory areas to execute voluntary movement in humans.

## Methods

### Subjects

Ten healthy right-handed volunteers participated in this study (ten males, mean age 32.6 ± 8.0 years). Each subject was in good health, and free of medication before the experiment. Informed consent for participation in the experiment, which was approved by the Ethics Committee of the National Institute of Technology (Nagoya, Japan), was obtained from all subjects. All experiments were performed in accordance with approved guidelines and regulations.

### Stimulation procedure

The right median nerve was stimulated at the wrist with a saddle type electrode. The electrode was fixed on the median nerve so as not to move during the recording. The cathode was placed 2 cm proximal to the anode. Constant current square wave pulses (duration, 0.2 ms) were provided, and the inter-stimulus interval was 500 ms. The stimulus intensity was adjusted to produce a slight twitch of the abductor pollicis brevis muscle.

### Experimental Procedure

Subjects were seated on a chair in a shielded room. They performed the four motor tasks using the right fingers. The tasks were as follow: 1). Ball Rotation Task (BR task): Rotation of two wooden balls (diameter 35 mm, weight 25 g each) as quickly and precisely as possible in a counterclockwise direction on the palm side of the right hand with eyes open (Fig. 1). This task required smooth coordinated movement of all fingers. Since no subjects had previous experience with this task and they first could not rotate two balls smoothly, they practiced this task before the recording. 2). Ball Grasping Task (BG task): Continuous grasping of two balls with all fingers of the right hand. The instruction was to grasp two balls with all fingers for about 10 sec and then to relax for about 2 to 3 sec. Subjects repeated this sequence of movements. 3). Stone and Paper Task (SP task): Subjects performed opening and closing right hands continuously at their own pace. 4). Air Rotation Task (AR task): Subjects moved their right fingers as if they were performing the BR task in the absence of two balls. During continuous electrical stimulation for SEFs, they performed four tasks. Although electrical stimulation of the median nerve produced small twitches of the abductor pollicis brevis muscle, they did not interfere with the movement of each task. We also recorded SEFs during the stationary state with two balls putting on the right palm with no finger movement (Stationary condition). The order of tasks in each condition was randomized among the subjects. The duration of each task was about 3 min. In each condition, we averaged 300 stimuli for SEF recordings. Subjects were instructed to concentrate on the finger movement and not to pay attention to the electrical stimulation during the experiment.

### Data acquisition and analysis

The MEG signals were recorded with a helmet-shaped 306-channel detector array (Vectorview, Elekta Neuromag Yo, Helsinki, Finland), which was comprised of 102 identical triple sensor elements. Each sensor element consisted of two orthogonal planar gradiometers and one magnetometer coupled to a multi-SQUID (superconducting quantum interference device) and thus provided three independent measurements of the magnetic fields. The MEG signals were recorded with a bandpass filter of 0.03–300 Hz and digitized at 1000 Hz. Epochs in which the signal variation was larger than 3000 fT/cm were excluded from the averaging. The period of analysis was from 100 ms before to 300 ms after the onset of the electrical stimulus. The data for 100 ms before the stimulus were used to calculate the baseline.

Prior to the MEG recording, four head position indicator (HPI) coils were placed at specific sites on the scalp. To determine the exact location of the head with respect to the MEG sensors, an electric current was fed to the HPI coils, and the resulting magnetic fields were measured with the MEG sensors. These procedures allowed for alignment of the individual head coordinate system with the MEG coordinate system. The locations of HPI coils with respect to the three anatomical landmarks (nasion and bilateral preauriculas) were also measured using a three-dimensional digitizer to align the coordinate systems of MEG with magnetic resonance images (MRI) obtained with a 3 Tesla MRI system (Allegra; Siemens, Erlangen, Germany). The x-axis was fixed with the preauricular points, the positive direction being to the right. The positive y-axis passed through the nasion and the z-axis thus pointed upward.

To identify the equivalent current dipoles (ECDs) in MEG components, sources of measured responses to the electrical stimuli were modeled with the time-varying current dipoles method^48^. The early SI response is estimated at around 20 ms in all subjects. In the period when clear dipolar magnetic field patterns were found, the ECDs that best explained the most dominant signals were determined by a least-squares search, based on 14 to 18 channels around the channel that had been used to measure the peak amplitude of RSS waveforms. The goodness-of-fit (GOF) value of an ECD was calculated to indicate in percentage terms how much the dipole accounted for the measured field variance. Only ECDs which accounted for more than 80% of the GOF in a channel subset were accepted. Then, all MEG channels were used to compute the time-varying dipole model allowing the strengths of the previously found ECDs to change over the entire period of analysis while the source location and orientations were kept fixed.

Three components were identified from the source waveforms in all subjects. The first component peaked at approximately 20 ms (M20). The second large deflection was at approximately 30 ms (M30). The final component was a small deflection peaking approximately 38 ms (M38). To compare the peak amplitude of ECD components among the stationary and four motor tasks, we first conducted a one-way repeated measures analysis of variance (ANOVA), with Task (BR, BG, SP, and AR task, Stationary condition) as the factor. To analyze the assumption of sphericity prior to the repeated ANOVA, we used Mauchly’s test of sphericity. A post-hoc analysis followed by the Bonferroni test was used for multiple comparisons. In addition, to investigate the relationship between the dexterity of finger movements and object operation in sensorimotor integration, we conducted a two-way repeated measures ANOVA, with Movement Complexity (Simple, Complex) and Object (Two balls, No object) as the factors. While the BR and AR tasks were complex finger movements, the BG and SP tasks were simple finger movements. Also, while the BR and BG tasks operated objects (two balls), the SP and AR tasks used no object. A post-hoc analysis followed by the paired t-test. The level of statistical significance was set at 5% (P < 0.05).
